# Treatment of Ekbom Syndrome With Clozapine and Electroconvulsive Therapy

**DOI:** 10.7759/cureus.30469

**Published:** 2022-10-19

**Authors:** Aakanksha Singh, Riya Shah, Rashmin Cholera, Punya Mulky

**Affiliations:** 1 Department of Psychiatry, Bhartiya Arogya Nidhi Hospital, Mumbai, IND

**Keywords:** electroconvulsive therapy (ect), ekbom syndrome, clozapine, schizophrenia, delusion of parasitosis

## Abstract

Delusion of parasitosis (DP), which is also known as Ekbom syndrome, is a delusional disorder characterised by a false, fixed belief of being infested by insects or mites, despite the lack of supporting medical evidence. This disorder presents most commonly with the “Matchbox sign.” DP can present as a primary or secondary delusional disorder. It can be associated with various psychiatric conditions such as schizophrenia spectrum disorders, mood disorders, anxiety or substance abuse. Several organic conditions such as dementia, malignancies, vitamin deficiencies and cerebrovascular accidents can mimic symptoms of DP. Hereby, we present a case of schizophrenia in a young woman associated with DP in our inpatient care and the treatment outcome with the use of clozapine and electroconvulsive therapy (ECT) regimen.

## Introduction

Delusion of parasitosis (DP) or Ekbom syndrome is a delusional disorder characterised by a false, fixed belief of being infested by insects or mites, despite the lack of supporting medical evidence [1,2]. Even though it is a psychiatric disorder but patients first present to a dermatologist or a primary care physician [2]. DP can present as a primary delusional disorder of the somatic type, or as a secondary functional psychiatric symptom of schizophrenia, major depressive disorder or bipolar disorder. In a few cases, this delusion is shared by family members or significant other, also known as folie à deux [3]. This disorder presents most commonly with the “Matchbox sign,” wherein a patient arrives at the doctor's office with items extracted from the skin as evidence of a parasitic infestation and are typically stored in a small container such as a matchbox [3-5]. Other organic conditions such as dementia, peripheral neuropathy, substance abuse (cocaine and amphetamine abuse), cerebrovascular accidents, malignancies or vitamin B-12 deficiencies can present with symptoms of DP [6,7]. Thus, highlighting certain differences, we present a case of schizophrenia in a young woman associated with a DP in our inpatient care.

## Case presentation

A 35-year-old woman came to our inpatient care appearing paranoid and aggressive towards the staff. The patient was a known case of schizophrenia and never received psychiatric care beyond a few weeks, due to her lack of insight. She had no past or family history of psychiatric illness.

The parents gave a history of mistrust towards them, refusal to eat food cooked by the mother; frequent use of foul words, poor self-care, extensive scratch marks and hyperpigmented lesions on both her hands. These symptoms started insidiously eight years ago, following the breakdown of her relationship and interpersonal disputes with her parents. Her course has been fluctuating, with multiple exacerbations in symptoms of suspiciousness, agitation, abnormal behavior, staying away from home for 2-3 years, and difficulty sustaining a job. The patient was started on antipsychotic medicines seven years back by their family physician, to which there was a partial response, as there was a reduction in her aggression and an improvement in her biological functions. She stopped the medications due to her poor insight within two months.

Over the last six months, the patient felt that mites on her bed were entering her body and causing her to itch incessantly. According to the patient, the mites crawled into the mattress in the night through a duct near the window of her room. She firmly believes that these mites are transmitted from the pigeon’s faeces which caused her itching leading to sleep disturbances at night. The patient slept on a different mattress in the living room and did not allow anyone to touch her bedding, clothes, or any other personal belongings. During the inpatient care, routine blood tests such as complete blood count, thyroid, kidney, and liver function test, and a urine drug screen was carried out and revealed no abnormalities.

On mental state examination (MSE), the patient appeared withdrawn, with a fearful mood and flat affect. Her thought process revealed delusions of reference, persecution and parasitosis. The patient denied any perceptual abnormalities and had no cognitive impairment. Her judgment was impaired. She refused to take medications and had very poor insight into her illness.

On physical examination, multiple pruritic rashes were characterised by hyperpigmentation and redness due to excoriations on her upper extremities. Despite frequent reassurances from her family denying the presence of mites entering her body, the patient's conviction was unshakeable. A dermatology consultation was carried out for the patient in the hospital. A diagnosis of extensive papule urticaria with post-inflammatory hyperpigmentation (PIH) and DP was made. For which the patient was started on levocetirizine (5mg) tablets, permethrin 5% ointment, and a combination of fusidic acid and betamethasone. Despite this treatment, the patient perceived no improvement in her urticaria at bedtime and continued to believe that she was infested by mites. 

A trial of oral psychotropics, starting with Pimozide 2mg twice daily and slowly incremented to 10mg per day in the next 4-6 weeks. There was no clinical change observed, Risperidone was started at 2mg twice a day and was increased to 16mg per day, and pimozide slowly cross-tapered and stopped. To this medication, the patient exhibited hand tremors and rigidity, which was treated with Trihexyphenidyl 2mg at bedtime. As a result of extrapyramidal symptoms, Olanzapine was started at 2.5mg twice daily and increased to 20mg per day. Risperidone was slowly cross-tapered and stopped. The patient tolerated this medicine well and appeared drowsy but no significant change in her behaviour or delusional beliefs in the next 6-8 weeks. Considering the long duration of psychosis and poor response to the above psychotropics, a clozapine regimen was initiated at 25mg/day, which was slowly up titrated to 600mg/day over the next 2-3 weeks. Also, a trial of electroconvulsive therapy (ECT) was given simultaneously. The patient was administered a total of 12 bitemporal ECTs, thrice a week. During inpatient care, the patient’s weight and blood parameters were closely monitored. There was gradual improvement in her aggression and paranoia, as well as her delusion remitted partially (only the idea persisted). After two months of discharge, the patient was regular with her medication and subsequent follow-ups.

## Discussion

DP is an uncommon disorder, and its prevalence remains unknown [5]. Most recent studies had a mean age of presentation for DP is roughly 50 years, occurring most commonly in women [1,2,5,8]. As opposed to this, our patient had an earlier age of onset. There is limited data on the epidemiological occurrence of DP [7]. Diagnosing a case of DP is challenging for physicians as the patient’s symptoms could go back months or even years and can be associated with comorbid psychiatric conditions or other organic conditions. Depression (74%) was the most common comorbid condition, followed by substance abuse (24%) and anxiety disorders (20%) [2,5]. Our patient presented with a long duration of schizophrenia associated with symptoms of DP. Delusion of persecution is the most common type of delusion occurring in schizophrenia, but our case presented symptoms of DP alongside symptoms of persecution and reference, which is a rare phenomenon. Many patients are hesitant to consult a psychiatrist due to the perceived stigma of mental illnesses. Also, they firmly believe they have a parasitic infestation, not a psychiatric condition. They are usually first treated by a general physician and a dermatologist. In nearly 12% of cases, family members or spouses share their delusional beliefs, leading to a delayed psychiatric consultation [3,5]. However, in this case, the patient's family had no symptoms of folie à deux.

Since DP can have multiple origins, taking a detailed history is crucial to rule out all other medical conditions and plan appropriate treatment. Persistent symptoms of delusions necessitate treatment with antipsychotics [7]. In various literature reviews, it was indicated that symptoms improved with pimozide, olanzapine, and risperidone after at least a few months of treatment [3,5]. In this case, the patient was unresponsive to the above antipsychotics even after adequate doses and 4-6 weeks of treatment duration. The initiation of clozapine treatment and the use of ECT in combination showed gradual improvement in symptoms of DP. Although several studies have reported the safety and effectiveness of ECT augmentation in clozapine-resistant schizophrenia, not much is reported about this combination for the treatment of primary or secondary cases of DP [9]. Only one case report used a combination of clozapine and escitalopram in a case of secondary DP [10], and another case report from India used a combination of ECT and chlorpromazine to treat DP in a young 28-year-old woman [11].

## Conclusions

This case report highlights the challenges faced in diagnosing and managing cases of delusion parasitosis because of the rarity of its presentation. Most patients have poor insight and close resemblance with multiple dermatological conditions. Due to this, the patient presents late to a psychiatrist. Unfortunately, there is not much literature on the successful treatment of DP. Treatment with clozapine and ECT may be effective in treating non-organic delusional parasitosis. However, future studies are needed for further evidence. In addition, a good rapport with the patient, timely intervention, regular follow-ups and good family support could prove beneficial in the management of DP.
